# The ability of magnetic field sensors to monitor feeding in three domestic herbivores

**DOI:** 10.7717/peerj.5489

**Published:** 2018-09-13

**Authors:** Christina C. Mulvenna, Rory P. Wilson, Nikki J. Marks, Aaron G. Maule, David M. Scantlebury

**Affiliations:** 1 School of Biological Sciences, Institute for Global Food Security, Queen’s University Belfast, Belfast, United Kingdom; 2 Biosciences, College of Science, Swansea University, Swansea, United Kingdom

**Keywords:** Data logger, Magnetometer, Accelerometer, Behaviour classification, Remote sensing, Food intake, Herbivore, Agriculture, Cow, Sheep

## Abstract

The rate at which animals ingest food is a fundamental part of animal ecology although it is rarely quantified, with recently-developed animal-attached tags providing a potentially viable approach. However, to date, these methods lack clarity in differentiating various eating behaviours, such as ‘chewing’ from ‘biting’. The aims of this study were to examine the use of inter-mandibular angle sensors (IMASENs), to quantify grazing behaviour in herbivores including cattle (*Bos taurus*), sheep (*Ovis aries*) and pygmy goats (*Capra aegagrus hircus*) eating different foodstuffs. Specifically, we aimed to: (1) quantify jaw movements of each species and determine differences between biting and chewing; (2) assess whether different food types can be discerned from jaw movements; and (3) determine whether species-specific differences in jaw movements can be detected. Subjects were filmed while consuming concentrate, hay, grass and browse to allow comparison of observed and IMASEN-recorded jaw movements. This study shows that IMASENs can accurately detect jaw movements of feeding herbivores, and, based on the rate of jaw movements, can classify biting (taking new material into the mouth) from chewing (masticating material already in the mouth). The biting behaviours associated with concentrate pellets could be identified easily as these occurred at the fastest rate for all species. However, the rates of chewing different food items were more difficult to discern from one another. Comparison of chew:bite ratios of the various food types eaten by each species showed no differences. Species differences could be identified using bite and chew rates. Cattle consistently displayed slower bite and chew rates to sheep and pygmy goats when feeding, while sheep and pygmy goats showed similar bite and chew rates when feeding on concentrate pellets. Species-specific differences in chew:bite ratios were not identified. Magnetometry has the potential to record quantitative aspects of foraging such as the feeding duration, food handling time and food type. This is of major importance for researchers interested in both captive (e.g., agricultural productivity) and wild animal foraging dynamics as it can provide quantitative data with minimal observer interference.

## Introduction

Food acquisition is pivotal to animal life, providing the necessary energy to power all processes (McMahon & Bonner, 1983; Schmidt-Nielsen, 1997) including growth (Vézina et al., 2009; Careau et al., 2013), locomotion (Parker, Robbins & Hanley, 1984; Schmidt-Nielsen, 1972) and reproduction (Rödel et al., 2015). However, even within a single species, food type and food availability vary with time and space (Massei, Genov & Staines, 1996; Ben-David, Flynn & Schell, 1997; Gibson, 2001; Cooke et al., 2006), which affects the rate of food ingestion. In addition, even after food is encountered, the rate at which it is ingested is determined by species-dependent morphological and behavioural attributes such as bite size, bite rate and handling time (Owen-Smith, 1992). In turn, these attributes vary with animal body mass (Shipley et al., 1994; Wilson & Kerley, 2003) and with the physical manifestation of the food, such as its structure and toughness (Balch, 1971; Trudell & White, 1981; Wilson & Kerley, 2003; Ribeiro et al., 2012).

Herbivores represent a particular case in studies of food ingestion because, in contrast to carnivores, they generally spend little time searching for food. Instead, their ingestion rates are primarily determined by bite rate (i.e., the rate at which food is taken into the mouth and subsequently swallowed) (Shipley, 1999) and bite size (the amount food taken into the mouth per bite) (Trudell & White, 1981; Gross et al., 1993). Bite rate generally follows some inverse relationship to bite size (Black & Kenney, 1984; Rode, Robbins & Shipley, 2001; Ribeiro et al., 2012) as larger bites require more processing (e.g., chewing) before the next bite can be taken (Gross et al., 1993). The process of food mastication in herbivores also varies with food type, with tougher foods requiring more processing (Balch, 1971; Bourne, 1977) and therefore presenting longer time periods between subsequent bites (Newman, Parsons & Penning, 1994). For studies wishing to determine ingestion rates of herbivores, therefore, the ability to differentiate various jaw movements as either biting (food acquisition) or chewing (processing, masticating) is critical for accurate estimations of food intake.

Previous studies seeking to determine food ingestion rates have used direct observations and/or video recordings of animals eating (Hanson & Defran, 1993; Doolan & MacDonald, 1996; Ruckstuhl, Festa-Bianchet & Jorgenson, 2003). However, such methods are prone to interruptions in observations due to the study species operating in complex, or light poor, habitats or simply being elusive. In addition, bite rates are difficult to determine accurately as mandible movements can be missed easily by the observer. Remote sensing systems have attempted to quantify ingestion rates using various animal-attached transducers to eliminate the need for visual observations (Penning, 1983; Penning, Steel & Johnson, 1984; Beauchemin et al., 1989; Matsui & Okubo, 1991; Abijaoudé et al., 1999; Desnoyers et al., 2009), which, though useful in detecting the initiation and duration of feeding, have encountered difficulties in classifying jaw movements into either biting (acquiring food) or chewing (processing/masticating food). Such equipment also tends to be bulky and poorly transferrable between different individuals. One relatively small transducer-based emerging technology with promise uses accelerometers (Watanabe et al., 2008; Naito et al., 2010; Iwata et al., 2012; Andriamandroso, Lebeau & Bindelle, 2015; Alvarenga et al., 2016) attached to the mandibles of subject animals to monitor jaw movements associated with food ingestion. However, thus far, this approach seems unable to quantify masses ingested (Viviant et al., 2010). A study by Rombach et al. (2018), employed the use of ‘RumiWatch System’ comprising an accelerometer and pressure sensor attached to dairy cattle via a head harness. From this, jaw movements could be detected reliably, but there were issues in the classification of jaw movements. In contrast, acoustic monitoring systems which used sounds to differentiate the “*ripping of biting*” and the “*grinding of chewing*” (Laca et al., 1992) have proved promising (Laca et al., 1992; Ungar & Rutter, 2006; Navon et al., 2013), but are nevertheless prone to interference from external sources (Navon et al., 2013).

Perhaps the most promising technology that may be used for determining food ingestion is based on magnetometry. This uses a magnet on the lower mandible and a magnetic field strength-measuring system on the upper mandible to document jaw movement *via* variation in perceived magnetic field intensity (Koga et al., 2001). Tested initially on laboratory mice (Koga et al., 2001), this method was first used to study the feeding behaviour of free-ranging animals on Magellanic penguins *(Spheniscus magellanicus)* after ground-truthing on captive penguins (Wilson et al., 2002). Here, individual prey items as well as their prey mass were determined using beak angle and other non-consumptive behaviours, such as preening, vocalizations and breathing were also observed (Wilson et al., 2002). This method was later applied to a number of mammal, bird and turtle species by Ropert-Coudert et al. (2004), in which information on the timing of prey intake as well as the amount and quality of food could also be estimated. Of particular note was that these authors used the oscillation in the sensor-perceived magnetic field data to classify biting and chewing in horses. Fossette et al. (2008), applied this technology to leatherback turtles, and, while they were able to determine beak openings, they could not isolate feeding behaviours. Overall, therefore, of the various methods used to examine food ingestion, magnetometry appears to show the greatest promise as a tool for use in ranging herbivores.

The aims of this study were to examine the use of animal-attached magnets and magnetic field sensors (Inter-Mandibular Angular Sensors—IMASENs, *sensu* Wilson et al. (2002)) to quantify grazing behaviour in herbivores of different sizes eating different foodstuffs. Specifically, we aimed to: (1) quantify jaw movements of each species and determine differences between biting and chewing; (2) assess whether different food types can be discerned by jaw movements; and (3) determine whether species-specific differences in jaw movements can be detected. Success in this venture should help workers define the quantities of foodstuffs taken by both wild and farmed animals as well as, if the ingestion of specific plant types can be ascertained, this could help understand how herbivores selectively impact the vegetation in their ecosystems.

## Methods

### Technology

The devices used consisted of a multi-channel logger (‘Daily Diary’—supplier Wildbyte Technologies: http://www.wildbyte-technologies.com/) which contained *inter alia* a tri-axial magnetometer working with 12-bit resolution (±1.3 Ga) at 13 Hz for cattle and 40 Hz for sheep and goats to supply information to a 32 Gb micro SD card (SanDisk^®^, USA). The system was powered by a 3.7 V lithium-ion rechargeable battery (4 × 2 × 0.5 cm). The complete system was housed in a rounded plastic housing (4. 5 × 4 × 1.8 cm) before being wrapped in waterproof insulating tape to avoid environmental damage. Total weight of the magnetic field sensor, case and battery was 36.5 g. We used a disk-shaped (2 cm dia. × 1 cm) neodymium boron magnet (0.46 Tesla (T); First4magnets^®^, Tuxford, UK) to provide a magnetic field detectable by the device.

#### Calibration

To determine if the magnetic field sensor could detect changes in the magnetic field caused by varying proximity of the magnet, we moved the magnet towards and away from the magnetometer by approximately 5 cm to simulate jaw movements of a feeding animal. This was repeated at distances of 15 cm, 30 cm and 45 cm to simulate deployment of the technology on animals of varying head size. This allowed us to approximate a maximum distance between magnet and sensor while still providing a clear signal indicative of feeding.

### Animals and logger attachment

**Figure 1:**
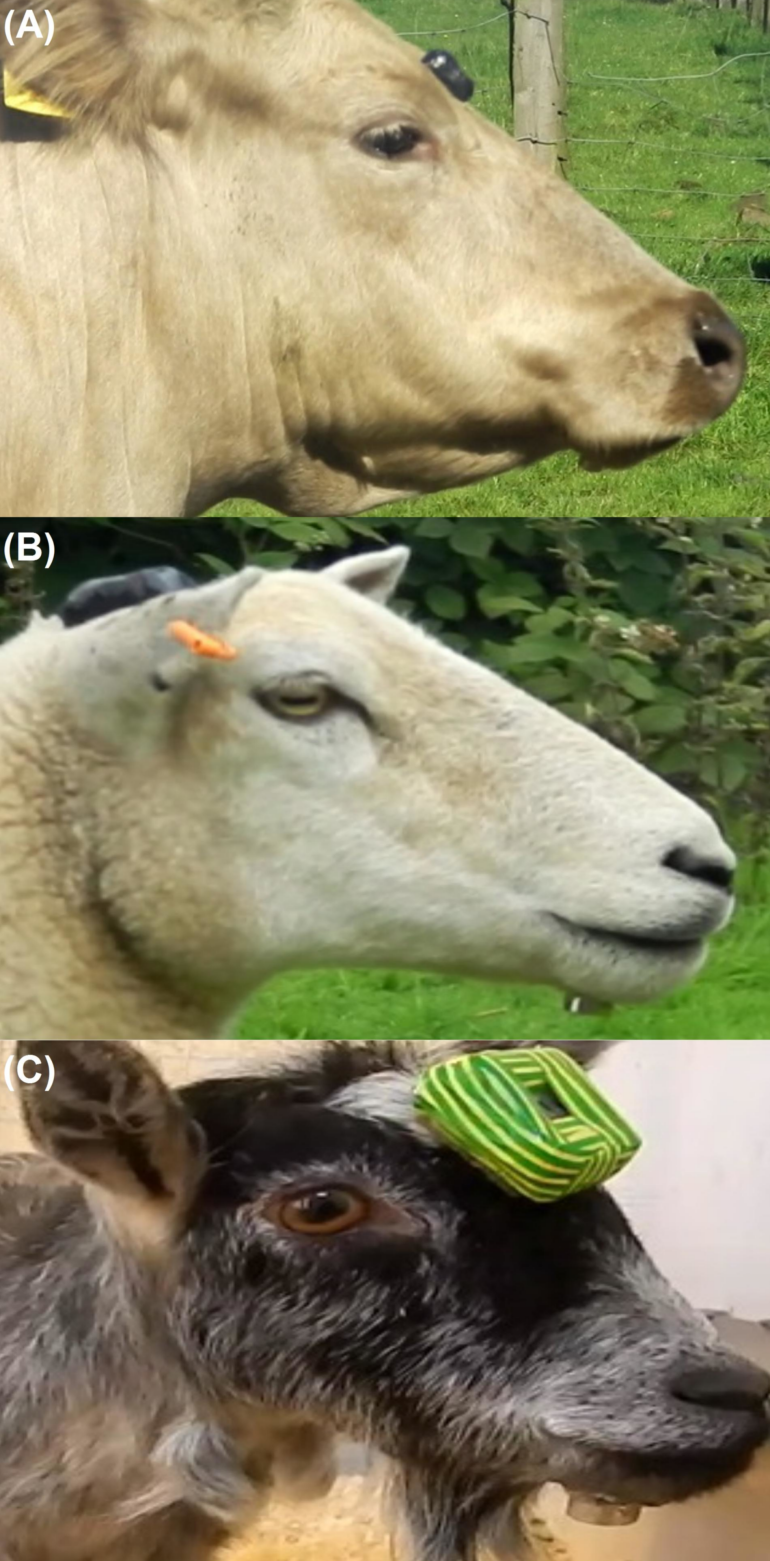
Sensors shown deployed on cows, sheep and pygmy goats. Attachment of magnetic field sensor and neodymium magnet (IMASEN) on each of the species used: (A) cow, (B) sheep and (C) pygmy goat. Credit C.C. Mulvenna.

Work was conducted at two sites in Northern Ireland between July and October 2016. Subjects included two adult cows (*Bos taurus*) and two adult ewes (*Ovis aries*) located in Carnlough (Co. Antrim), as well as two female, adult pygmy goats (*Capra aegagrus hircus*) in Rasharkin (Co. Antrim). The study was conducted on one focal animal at a time. To attach devices, cattle were restrained in a cattle crush, whilst sheep and pygmy goats were restrained manually. The magnet was attached to the underside of the mandible at the most anterior point and the IMASEN attached to the frontal region of the head of each animal using adhesive (Impact Adhesive, Evo-stik/Bostik La Défense, Paris, France) (Fig. 1). Distances between magnetic field sensor and magnet varied between species due to differences in head size (Table 1). While there is some concern that magnetic fields may influence animal behaviour (Ernst & Lohmann, 2018; Vargová et al., 2018), previous studies which have employed this method have noted no obvious effects of magnetic fields (Wilson et al., 2002; Ropert-Coudert et al., 2004). Nevertheless, we compared the grazing behaviour of sheep with and without magnets and sensors attached. Devices remained on the animals for approximately two hours. Animals were recaptured after the study and both the magnetometer and magnet were removed easily using scissors to clip the hair to which the devices had been glued. Data were then downloaded from the IMASEN. All animals used were in good health and were not deprived of food before the study. Ethical permission was granted by the Ethical committee, School of Biological Sciences, Queen’s University Belfast.

**Table 1:** Comparison of the feeding behaviour of different herbivores ingesting different foodstuffs using the IMASEN, including data on the recording frequency used and the mean distance between magnet and magnetic field sensor. Details of data collection and food provision to each species with observed median bite and chew rates. Recording frequency (Hz) of the IMASEN and mean distance (cm) between sensor and magnet (+SD) from the trials with details of the food types provided to each species (√ indicates ‘provided with’, × not provided with). The 95% confidence intervals of bite.rate min^−1^ and chew.rate min^−1^ are 95% CI.

**Species**	**Recording frequency (Hz)**	**Mean distance (cm)**±**SD**	**Concentrate**	**Grass**	**Browse**	**Hay**	**Bite.rate min**^−1^**(95% CI)**	**Chew.rate min**^−1^**(95% CI)**
*Cattle(Bos taurus)*	13	28 ± 3.1	√	√	×	×	85 (70 to 140)	73 (45 to 109)
*Sheep (Ovis aries)*	40	25 ± 10.3	√	√	√	×	242 (147 to 337)	181 (115 to 300)
*Pygmy goats(Capra aegagrus hircus)*	40	14 ± 0.39	√	×	×	√	303 (150 to 411)	164 (112 to 396)

### Feeding

Following device attachment, each subject animal was offered a range of foodstuffs (Table 1) and observed closely. Foodstuffs included concentrate pellets, approx. 20 mm length and 2 mm in diameter (Thompsons Feeding Innovation, Belfast) in a trough, grass in fields (2,840 m^2^ and 321 m^2^ for the cattle and sheep, respectively) which animals could freely graze on, and sycamore (*Acer pseudoplatanus)* leaves from small branches cut and offered to the animals *ad libitum* for the 15-minute measurement period. Since the pygmy goats were housed indoors, hay (instead of grass) was provided as food. Video recordings were taken of each animal eating each food type using a camera (Nikon^®^ Coolpix L820; Nikon Inc., Tokyo, Japan) at 30 fps with recordings lasting between 15 and 20 minutes. Videos were time-stamped so that they could be synchronised with IMASEN data.

### Video analysis

Each video was processed frame by frame using Avidemux 2.6, (32 bit) software. From each video and each animal, the duration of each feeding bout, the number of jaw movements per feeding bout and whether the subject was biting or chewing (Table 2) were recorded. Data were then combined with the IMASEN data, taking care to examine the extent to which the number of jaw movements matched waveforms in the data (although periods with <2 contiguous bites/chews were discounted).

**Table 2:** Definition of the terms used in this manuscript. Definitions used to classify jaw movements as either biting or chewing including calculations used to determine bite rate and chew rate. Where bite.min^−1^ is bite rate, chew.min^−1^ is chew rate with, time in in seconds.

**Action**	**Definition**	**Reference**
Bite	Grasping and removal of food using mouth	Chambers, Hodgson & Milne (1981)
Chew	Single dorso-ventral jaw movement to masticate food present in the mouth	Penning, Steel & Johnson (1984), Gross et al. (1993)
Bite.min^−1^	Number of bites per minute $ \frac{\text{No. bites}}{\text{time}} \times 60$	
Chew.min^−1^	Number of chews per minute $ \frac{\text{No. chews}}{\text{time}} \times 60$	

### Statistical analysis

Analyses were carried out in R Studio (R Core Team, 2018). Data were first examined for normality using Shapiro–Wilk tests and histograms were plotted. All data displayed non-normal distributions so non-parametric analysis were carried out. Specific analyses are outlined below.

#### Comparing methods to monitor jaw movements

To evaluate the reliability of the magnetic field sensor method, the numbers of jaw movements from magnetic field data were compared to those from video recordings for each species using interclass correlation coefficient estimates (ICC). ICC estimates and their 95% confidence intervals (95% CI), were calculated using the “irr” package, based on a single rater, absolute-agreement, two-way mixed-effects model (Gamer et al., 2012). Levels of agreement were deemed poor if *ICC* <0.5, moderate *ICC* = 0.5–0.75, good *ICC* = 0.75–0.9 or excellent *ICC*>0.9 ( Koo & Li, 2016).

#### Differences in rates of biting and chewing

Mann–Whitney *U* tests were conducted to determine if biting and chewing could be differentiated based on rates obtained from magnetic field data for each species using rate as the dependent variable and bite and chew as groups.

#### The effect of food type on bite and chew rate

To examine if bite and chew rates differed depending on of the food item being consumed for each species, permutations using the package “ez” (Lawrence, 2016) were conducted. These included the rate per minute (of biting and chewing) as the dependent variable, food type as the independent variable, and individual identification as a random factor to control for repeated measures. The number of permutations conducted was 1000. To determine if there was any variation in the chew:bite ratio due to food type, the median chew rate was divided by the corresponding median bite rate for each food item. This provided a measure of the number of chews conducted per bite of food. For cattle, the chew:bite ratio of concentrate and grass was compared using a Mann–Whitney *U* test. This test was also used to compare the chew:bite of concentrate and hay for pygmy goats. The chew:bite ratios of sheep eating concentrate, grass and browse were examined using Kruskal–Wallis tests.

#### Species differences in feeding rate as a function of food type

Comparison of the rates of biting and chewing of species eating the same food item could only be conducted on the food types; “concentrate pellets” and “grass”, because these food items were the same for more than one species. First, to compare bite and chew rates of cattle, sheep and pygmy goats feeding on concentrate, a generalised linear mixed model (GLMM) was conducted using the “lme4” package (Bates et al., 2015) (Table 3; model 1b). A GLMM was also used to compare of the rates of biting and chewing of cattle and sheep feeding on grass (Table 3; model 2b). Pygmy goats were excluded as they were not observed feeding on grass. *Post-hoc* analyses were conducted using Tukey adjustments using the “lsmeans” package ( Lenth, 2016). All statistics were deemed significant if *p* < 0.05. All graphs were produced using “ggplot” ( Wickham, 2009). To investigate if the chew:bite ratio varied between species, ratios of cattle, sheep and pygmy goats feeding on concentrate were examined using a Kruskal–Wallis test. A Mann–Whitney *U* test was used to compare the chew:bite ratios of cattle and sheep eating grass.

**Table 3:** Statistical details of the comparisons made in the text. Statistical refinement of GLMM models to identify differences in rate of biting and chewing as a function of species and food type. ∗ indicates an interaction and + indicates a main effect. AIC represents the Akaike information criterion value, *X*^2^ is the chi-squared statistic, *df* is the degrees of freedom and *p* is the probability value. “Individual” was included at the random factor within each model.

**Model No.**			**Dependent variable**	**Independent variable**	**AIC**	*X*^2^	*df*	*p*
1a			Rate min^−1^	Species ∗ Food type	157.99			
1b			Rate min^−1^	Species + Food type	157.24	3.24	2	0.19 (Final model)
1c			Rate min^−1^	Species	311.87	156.63	1	<0.001
1d			Rate min^−1^	Food type	164.99	11.75	2	<0.01
2a			Rate min^−1^	Species ∗ Food type	42.27		6	
2b			Rate min^−1^	Species + Food type	40.27	1 × 10^−4^	5	0.99 (Final model)
2c			Rate min^−1^	Species	52.74	14.47	4	<0.001
2d			Rate min^−1^	Food type	49.73	11.46	4	<0.001

## Results

Animals used in this study showed no disruptions in feeding behaviour once they were instrumented with the IMASEN: The feeding behaviour of sheep instrumented with IMASEN sensors and magnets was compared with non-instrumented sheep eating grass using video count data and no differences were observed in bite rate (*U* = 149.5, *p* = 0.913, 95% CI [−27.88 −27.33]) or chew rate (*U* = 682.5, *p* = 0.866, 95% CI [−23.79 −31.21]). We therefore concluded that the measurement technique had minimal effect on the way that our animals were observed to graze. Animals made no attempts to remove devices during periods of observation. The total length of recordings for each species was 47 minutes for cattle, 60 min for sheep and 49 minutes for pygmy goats. From this, 887 examples of biting and chewing were recorded in over 70 minutes of magnetic field data across species. Of these, 79 belonged to cattle, 241 were of sheep and 567 were of pygmy goats. The shortest instance of feeding occurred in pygmy goats, which was 0.2 seconds, during which two chews were completed; the longest instance of biting behaviour occurred over 201 seconds during, which a cow was observed to complete 372 bites.

### Evaluating the use of magnetic field sensor

**Figure 2:**
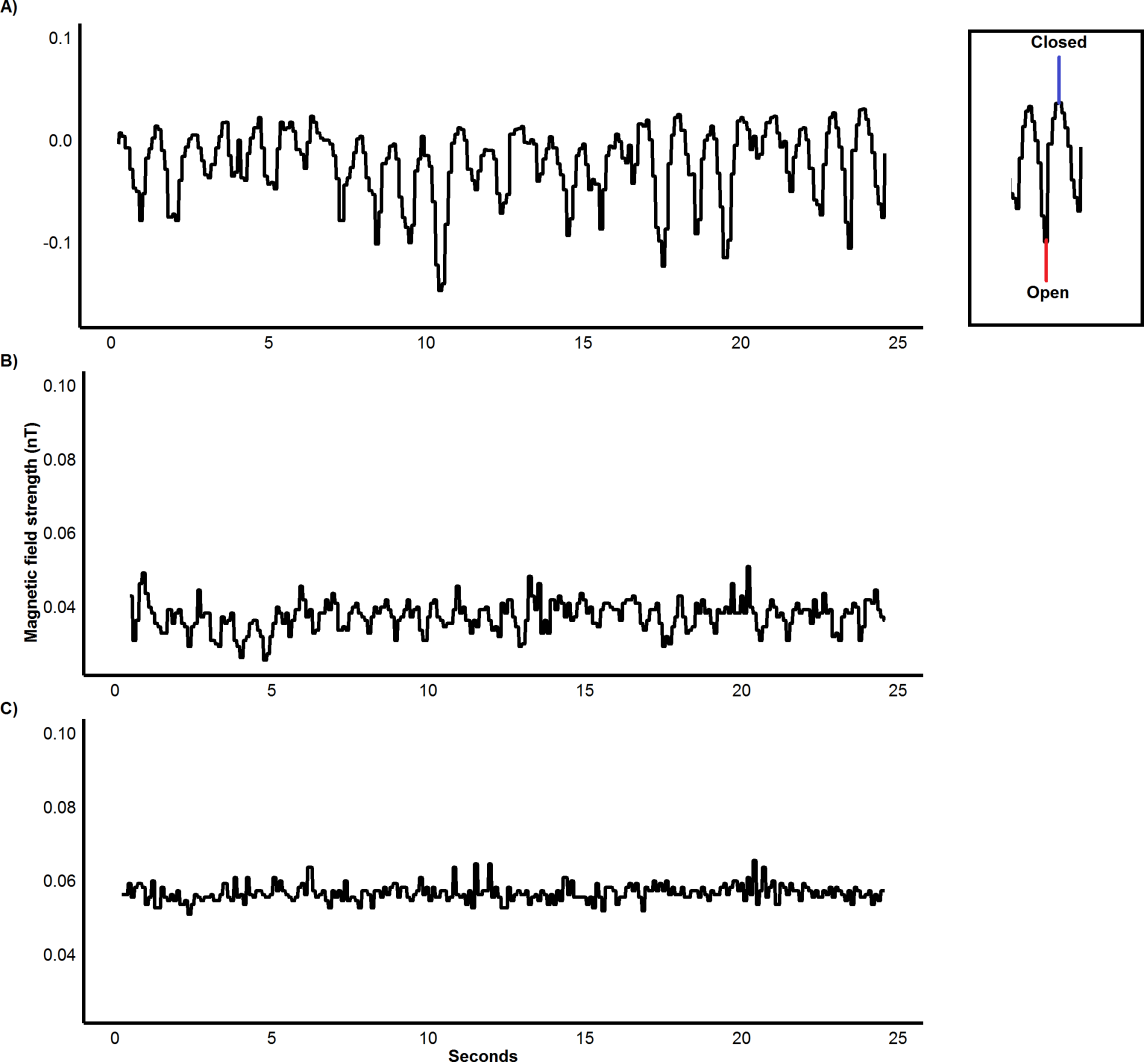
Variation in magnetic field strength with distance from magnet. Calibration of IMASEN: Differences in magnetic field strength, nanotesla (Nt), as a neodymium magnet (0.46 T) is moved 5 cm at the varying proximities of (A) 15 cm, (B) 30 cm and (C) 45 cm over 25 seconds.

Calibrations showed that oscillations in the magnetic field data caused by changing proximity of the magnet were clearly identifiable at magnet-sensor distances of 15 cm and 30 cm. However, oscillations were not distinguishable at distances of 45 cm (Fig. 2). The magnetic field sensor accurately measured the number of jaw movements in all species as shown by the high levels of agreement between sensor and video recordings (cattle, *ICC* = 1, 95% CI [0.99–1], sheep, *ICC* = 0.99, 95% CI [0.99–0.99] and pygmy goats, *ICC* = 0.99, 95% CI [0.99–0.99]).

**Figure 3:**
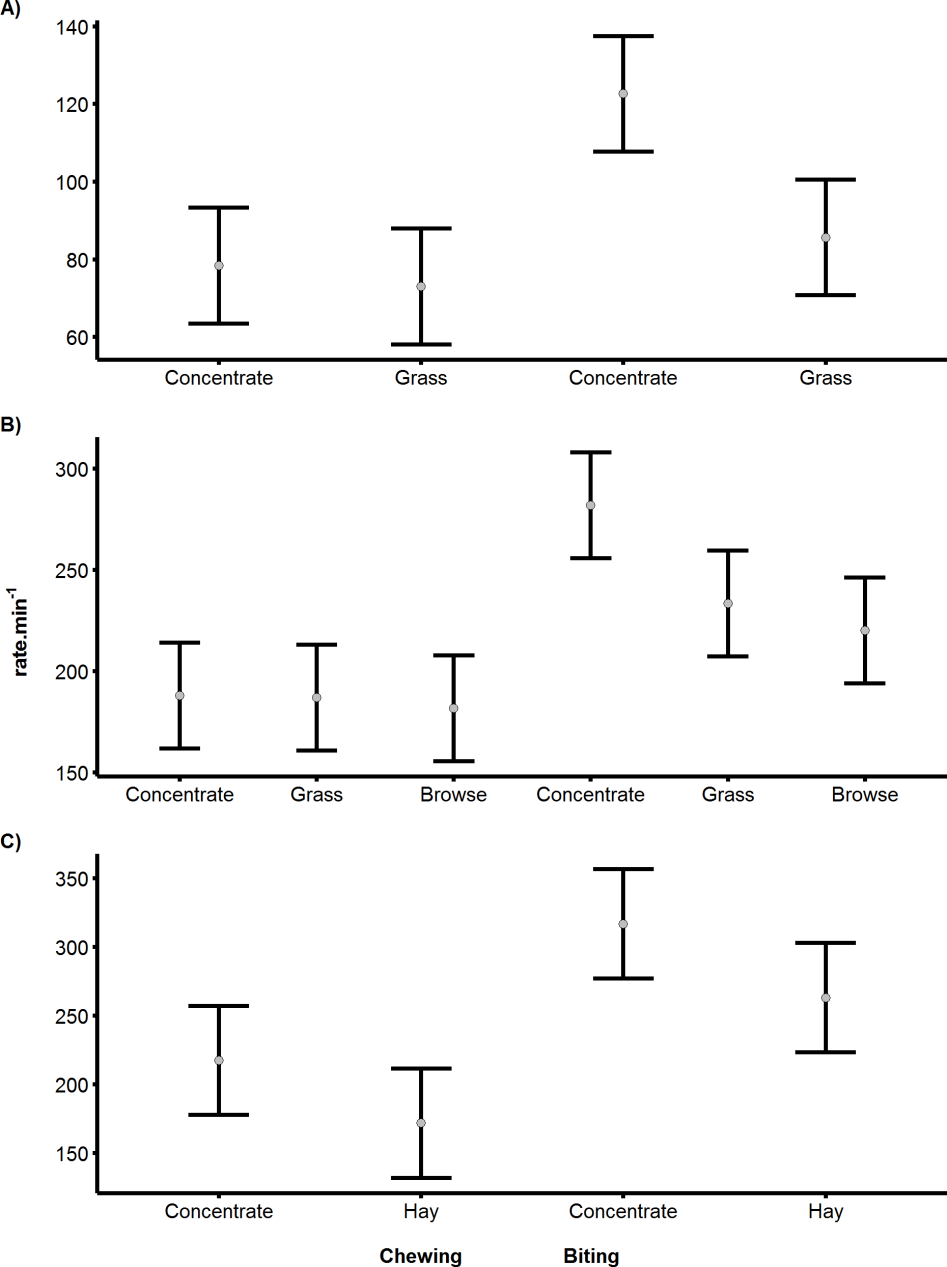
Differences in biting and chewing rates for cows, sheep and pygmy goats consuming different food. Differences in the mean rate.min^−1^ of biting or chewing depending on the food resource consumed by (A) Cattle, (B) Sheep (C) Pygmy goats. Error bars indicate differences according to Fisher’s LSD.

### Comparison of bite and chew rate

The median bite rates in cattle were 16% higher than the median chewing rates (*U* = 1,075, *p* < 0.001, 95% CI [8.64–28.86]). In sheep, biting occurred 33.7% faster than chewing (*U* = 61,733, *p* < 0.001, 95% CI [100.47–129.94]). The greatest difference between the bite and chew rates (Table 1) was observed in pygmy goats as the median bite rates observed were 84% faster than the median chewing rates (*U* = 10,456, *p* < 0.001, 39.52 to 71.38 95% CI [100.47–129.94]).

### Differences in bite and chew rate as a function of food type

The rates of biting and chewing were found to differ significantly between food types in all species (cattle *p* < 0.05, sheep *p* < 0.05 and pygmy goats *p* < 0.05). Specifically, the biting of concentrated pellets could be clearly identified, producing the highest mean bite rates recorded of 122, 266 and 317 bites.min^−1^, for cattle, sheep and pygmy goats, respectively (Fig. 3). However, in pygmy goats, chewing concentrate occurred at a rate that was 48% slower than the rate at which concentrate was bitten. Although there was an 11 bite.min^−1^ difference between the mean grass bite rate and browse bite rate in sheep, the difference was not significant (Fig. 3B). Examinations of chewing rates in cattle and in sheep showed no difference with food type. However, pygmy goats appeared to chew concentrate faster than hay (with a 49 chews.min^−1^ difference), this was not significant (Fisher’s least significant difference (indicated by the error bars)) (Fig. 3C). No significant difference was noted between the chew:bite ratios of cattle feeding on concentrate pellets or on grass (*U* = 0, *p* = 1). A similar result was found after comparison of the chew:bite ratios of pygmy goats when feeding on concentrate pellets and hay (*U* = 0, *p* = 1). Differences in the chew:bite ratios of sheep feeding on concentrate pellets, grass and browse could not be determined statistically as the ratio of chews to bites for each food item was the same (Table 4).

**Table 4:** Ratios of the chews required for bites of each food type for all species. Ratios of the number of chews to number of bites observed of each food type for each species.

**Species**	**Chew:Bite**	**Food type**
Cattle	0.6	Concentrate pellets
*(Bos taurus)*	0.9	Grass
Sheep	0.7	Concentrate pellets
*(Ovis aries)*	0.7	Grass
	0.7	Browse
Pygmy goat	0.6	Concentrate pellets
*(Capra aegagrus hircus)*	0.7	Hay

### Species comparisons of bite and chew rates

**Figure 4:**
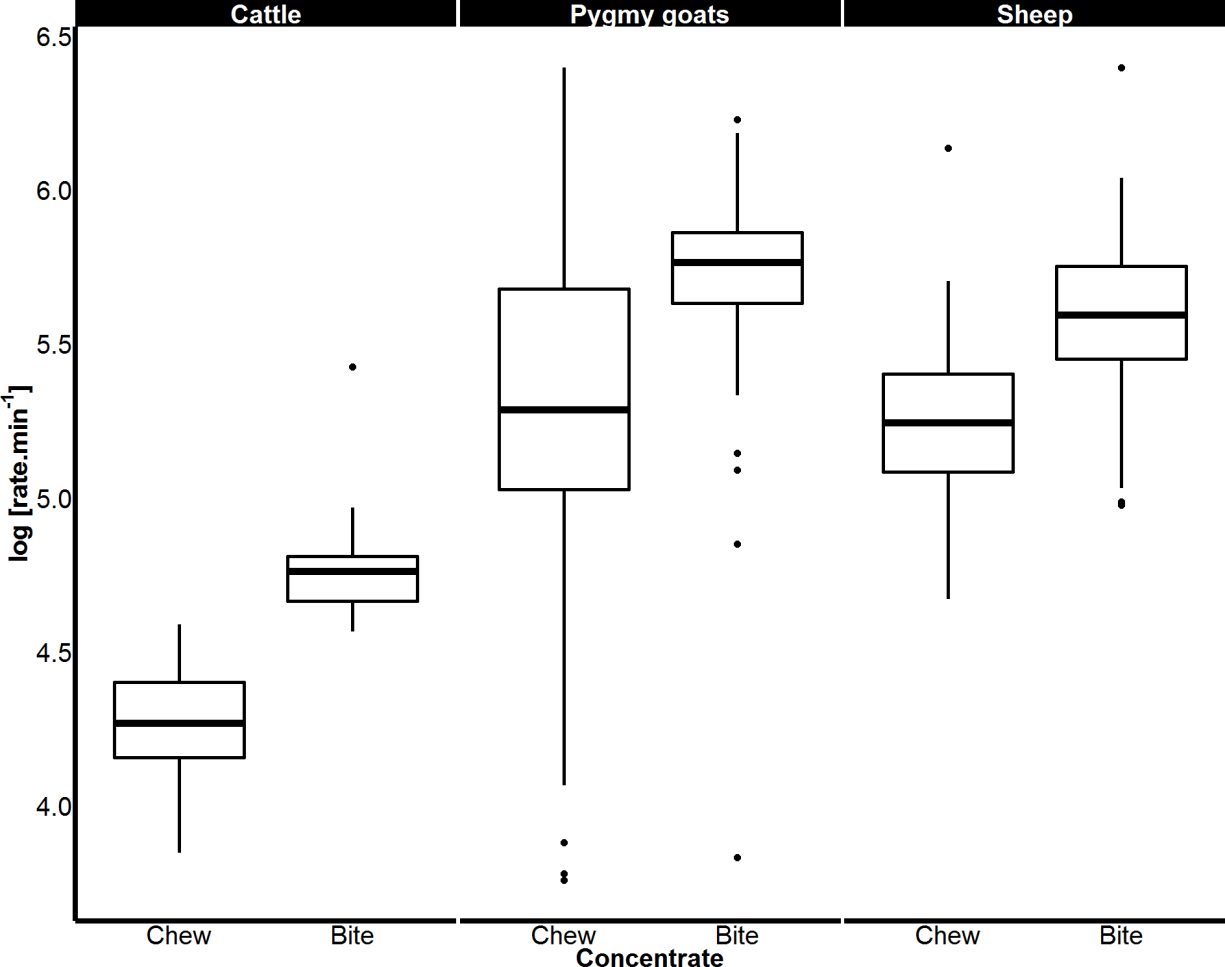
Variation in biting and chewing rates of cattle, sheep and pygmy goats eating concentrate pellets. Median bite rate and chew rate displayed by each species when feeding on concentrate pellets determined from a magnetic field sensor. Boxes represent the 25th and 75th percentiles; bars represent minimum and maximum rates.

**Figure 5:**
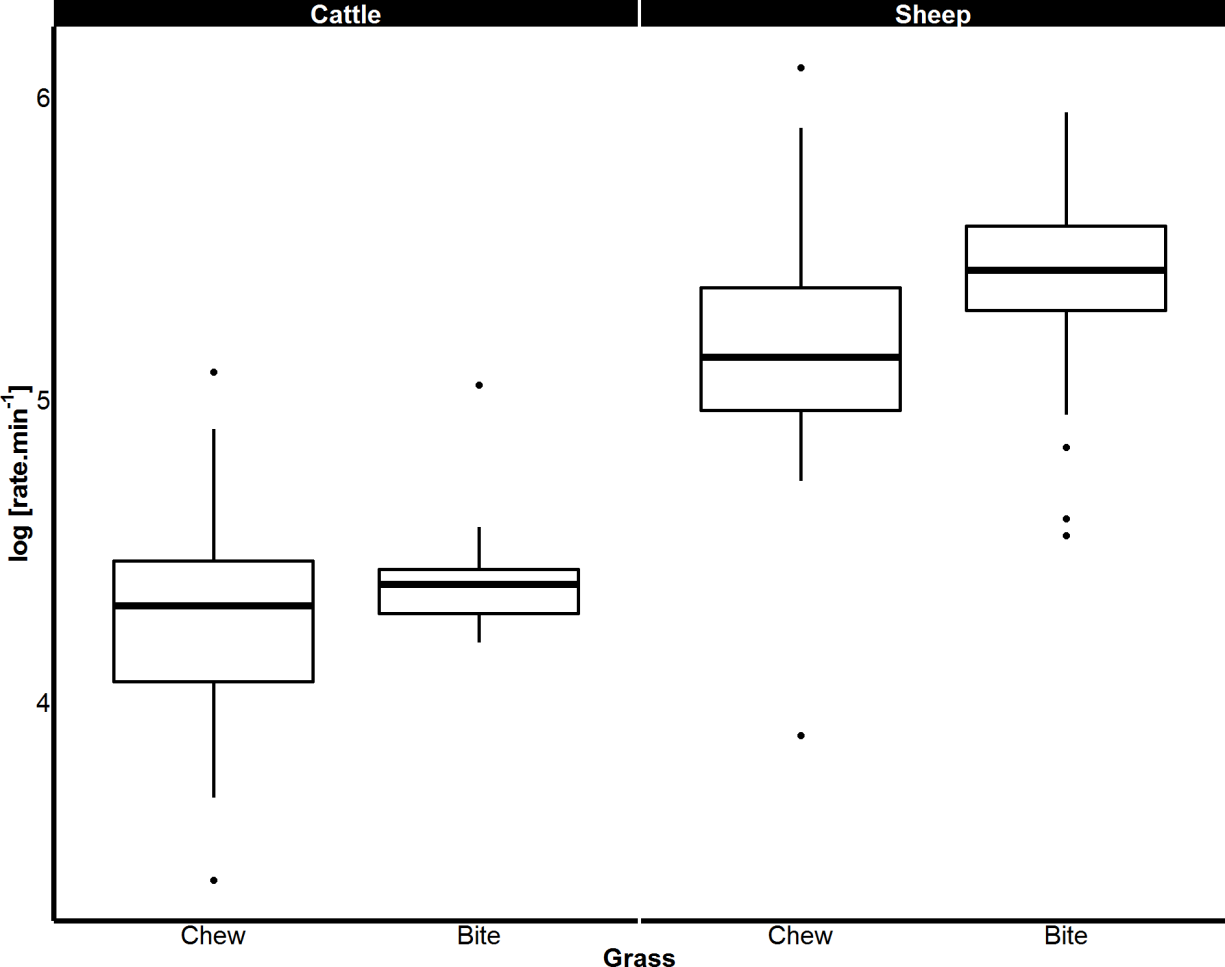
Differences in biting and chewing rates of cattle and sheep eating grass. Median bite rate and chew rate displayed by cattle and sheep when grazing on grass determined from magnetic field sensor. Boxes represent the 25th and 75th percentiles; bars represent minimum and maximum rates.

When feeding on concentrate, the rates of both biting and chewing displayed by sheep (biting *p* < 0.001; chewing *p* < 0.001) and goats (biting *p* < 0.001; chewing *p* < 0.001) were over twice those of cattle. Sheep and pygmy goats bit and chewed concentrate at similar rates (Fig. 4). When eating grass, sheep bit and chewed at rates that were 184% and 130% faster than cattle respectively (*p* < 0.001 for both, Fig. 5). Similar chew:bite ratios were shown by cattle, sheep and pygmy goats feeding on concentrate pellets (*H* = 2, *df* = 2, *p* = 0.36). Similar chew:bite ratios were observed in cattle and sheep feeding on grass (*U* = 1, *p* = 1, Table 4).

## Discussion

The basic premise behind this work is that accurate measurement of animal jaw movements during feeding can be used to derive feeding rates (Wilson et al., 2002). Such information can be used in a suite of important applied and blue skies issues, ranging from conservation efforts to production and welfare of animals in agricultural systems. Various methods have been applied to look at feeding rates, including pneumatic tubing, and various types of transducers such as accelerometers have been deployed on subject animals. Noted disadvantages of the previous systems include the inability to transfer equipment between species varying in size or misclassification of feeding jaw movements (Penning, 1983; Penning, Steel & Johnson, 1984; Beauchemin et al., 1989; Matsui & Okubo, 1991; Abijaoudé et al., 1999; Desnoyers et al., 2009). Below we consider how our work relates to those of others.

### Attachment of devices

A particular concern related to the attachment of magnets to animals involves the observation that some species may use the earth’s magnetic field for orientation (Lohmann & Lohmann, 1996; Holland et al., 2006). Although this is a complex issue, many studies, e.g., that by Mouritsen et al. (2003) on waved albatrosses (*Phoebastria irrorata*), or work on penguins (Koga et al., 2001; Wilson et al., 2002; Ropert-Coudert et al., 2004), have found no effect of magnet attachment on movement and navigation. Similarly, in our study, we observed no disruptions in behaviour or even attempts to remove devices or magnets. Indeed, we suggest that the relatively small size of the system compared to the body size of many herbivores, means that the approach should be useful for examining the foraging behaviour free-ranging species of various size. We recognise, however, that the IMASEN requires the recapture of the animal to obtain the data, which may be difficult for some species.

### Detection of jaw movements

The IMASEN employed in this study accurately determined the initiation and duration of feeding bouts and even quantified jaw movements well, with an excellent level of agreement (99–100%) between video recordings and magnetometers across species and food types. In addition, we suggest that the approach has potential for monitoring jaw movements not associated with feeding, such as vocalisations, grooming and breathing, as exemplified by Wilson et al. (2002) for penguins. However, care should be taken in the attachment of the sensor and magnet to ensure they are in close enough to produce clear oscillations in magnetic field strength as a result of the opening and closing of the jaw. For the strength of the magnet used within this study, we would recommend a maximum distance of 30 cm (Fig. 2). For different-sized study animals, variation in magnet size and strength coupled with the variable location of the magnetometer, if necessary through use of cables (Wilson et al., 2002), should give considerable flexibility to maximize the signal-to-noise ratio.

### Differentiating biting and chewing

A critical issue in determining food ingestion relates to the classification of biting *versus* chewing (Chacon, Stobbs & Sandland, 1976). Although the use of signal amplitude in sensor-perceived magnetic field strength should theoretically help in such classifications because it should reflect the varying distance between upper and lower jaw (Fig. 2), we found this difficult to assess because we had no specific protocol to calibrate the signal with jaw angle (cf. Wilson et al., 2002). Such a process would be difficult in large herbivores and it proved impossible to standardize magnet and sensor positioning both between species and between individuals (Table 1). Instead, therefore, we compared the wavelength of the oscillations in jaw movements to differentiate biting and chewing, as has been documented by Ropert-Coudert et al. (2004) for horses. Similarly, Mezzalira et al. (2014) used the same method to classify biting and chewing in cattle. Indeed, both studies describe a bite (means of 1.33 s in horses and 1 s, cattle) as taking more time than a chew (0.62 s, horses and 0.68 s, cattle). Curiously though, these results are at odds with ours, which clearly showed that bites took significantly less time than chewing in all species studied. This variation could be attributed to various factors listed previously including body size, food structure and density and certainly warrant further attention. That apart, the successful identification of biting and chewing shows the potential of this method to allow for more accurate estimates of animal feed intake as not all jaw movements result in the intake of food.

### Identifying differences due to food items

Previous studies investigating what animals are feeding on, and in what quantities, with a wider aim of investigating of animals on the environment have included methods such as stomach content analysis and scat sampling (e.g., Bird et al., 2012). Here, we suggest magnetic field sensors can be used to distinguish various food items being eaten based on biting and chew rates and chew:bite ratios. The biting behaviours associated with concentrate pellets could be easily identified from the other food items as these occurred at the fastest rate for all species; indeed, pellets may be more easily consumed due to their loose structure. However, there was some difficulty in distinguishing grass from leaves in sheep and, overall, no apparent differences in the rates of chewing of any food item eaten by any species. We also attempted to determine if the number of chews required per bite differed due to food type; again no differences were evident (Table 4). Although these results are preliminary, we recognise that jaw movement rates and processing requirements can vary depending on a number of factors, not least the ‘fibrousness’ of the food type. In this regard, where finer differentiation of food type is required, the simultaneous measurement of other factors such as jaw angle (Wilson et al., 2002; Ropert-Coudert et al., 2004), acoustics (Laca et al., 1992; Ungar & Rutter, 2006; Navon et al., 2013) and length of time engaged in chewing (Balch, 1971) could prove useful in identifying the food item being consumed. We hope that all this may be considered together to derive useful indices of vegetation types consumed by different herbivores. We note that advances in animal-attached technology is now enabling ever finer resolution of animal behaviour, including the incidence of biting in herbivores (Di Virgilio et al., 2018). It remains to be seen the extent to which enhanced consideration of jaw angle (perhaps vertically and horizontally using properly calibrated tri-axial magnetometers (Williams et al., 2017)) over time may provide cues as to vegetation type, and thereby a proper measure of rates of energy gain according to the landscape characteristics (Di Virgilio et al., 2018). Although this is important for farmed herbivores, it also has implications for studies on wild animals where GPS-type data may provide location and the IMASEN could reveal the details of vegetation choice (see Frank, Wallen & White, 2016 and references therein), not least because such choice informs ecologists about how it structures the plant community (De Vries et al., 2018). This is important in native species but may become critical when considering invasive species (see e.g., (Davis et al., 2016) and references therein).

### Species differences in rate of feeding

The comparison of rates of biting and chewing between species revealed that cattle fed consistently more slowly, at less than half the rates observed for both sheep or pygmy goats, regardless of food type. This is consistent with the idea that bite rate and chew rate decrease with increasing body size and bite size (Shipley et al., 1994; Wilson & Kerley, 2003), as the rate at which they can select and masticate bites is reduced (Druzinsky, 1993; Gerstner & Gerstein, 2008). Given allometric scaling issues, we suggest that examination of bite and chew rate data for sympatric competing individuals in the wild might serve to define food ingestion performance limits and indicate where different food types might favour one species over another (Illius & Gordon, 1987; Murray & Illius, 2000). The species studied displayed similar processing efforts with regards to the ratios of chews to bites when eating similar food types although our study was preliminary. We suggest that further investigation into other factors including bite size and/or handling time may identify more behaviours associated with different species. Variation in the rates of biting and chewing observed will also presumably be influenced by sample size, noting that we have included data of two individuals from each species. Thus, while we have described our results within the context of the feeding rates of cattle, sheep and pygmy goats in this study, we understand that these are not representative how the entirety of each species behaviours. Against this, we have demonstrated that magnetic field sensors can be applied to a range of species easily. Future studies with larger sample sizes can use this method to look in detail at true inter- and intra-specific feeding behaviours, which can be used to better understand animal food requirements, food competition or an animal’s impact on the environment.

## Conclusion

Overall, this study indicates that the application of a magnetic field sensor paired with a magnet has considerable promise as an approach to study the feeding behaviours of ungulates. Although our tests provided accurate estimates of feeding periods, jaw movements and feeding rates, and highlighted species differences in feeding behaviours, further work is required to refine the method to determine the specifics of the food item being consumed from the data. Once achieved, this should provide pivotal data on the foraging behaviour of free-ranging species according to food type and availability. We also recognise the potential of this system to study jaw movements which may not be associated with feeding such as social behaviours like vocalisations and grooming.

##  Supplemental Information

10.7717/peerj.5489/supp-1 Data S1 Raw data used for analyses

## Additional Information and Declarations

### Data Availability

The following information was supplied regarding data availability: The raw data are provided in a Data S1.
